# Mediating roles of corneal biomechanical and topographic parameters in eye rubbing and keratoconus based on the Chinese keratoconus cohort study

**DOI:** 10.3389/fbioe.2025.1595671

**Published:** 2025-06-26

**Authors:** Kaili Yang, Runqi Tu, Liyan Xu, Yuwei Gu, Qi Fan, Shanshan Yin, Yi Yuan, Anqi Chang, Yifan Wang, Chenchen Yin, Yonghao Zang, Chenjiu Pang, Daniela Oehring, Yibin Hao, Shengwei Ren

**Affiliations:** ^1^ Henan Provincial People’s Hospital, Henan Eye Hospital, People’s Hospital of Zhengzhou University, Henan University People’s Hospital, Zhengzhou, China; ^2^ Eye Institute, Henan Academy of Innovations in Medical Science, Zhengzhou, China; ^3^ Zhengzhou University People’s Hospital, Henan Provincial People’s Hospital, Henan Eye Hospital, Zhengzhou, China; ^4^ Henan University People’s Hospital, Henan Provincial People’s Hospital, Henan Eye Hospital, Zhengzhou, China; ^5^ Xinxiang Medical University, Henan Provincial People’s Hospital, Henan Eye Hospital, Zhengzhou, China; ^6^ School of Health and Human Sciences, University of Plymouth, Plymouth, United Kingdom

**Keywords:** eye rubbing, keratoconus, stiffness parameter at the first applanation, maximal corneal keratometry, mediation

## Abstract

**Purpose:**

Studies have shown that eye rubbing is associated with increased risk of keratoconus (KC). However, the potential mediating roles between eye rubbing and KC remain largely unknown. Hence, this study aims to explore the mediating roles of two specific factors, namely, the inverse of the stiffness parameter at the first applanation (-SPA1) and maximal corneal keratometry (Kmax) values, in the relationship between eye rubbing and KC.

**Methods:**

A total of 395 patients with KC and 396 controls from the Chinese keratoconus (CKC) cohort study were included in this case–control analysis. The Spearman correlation and generalized linear regression models were used to analyze the associations between the time of eye rubbing, -SPA1, Kmax, and KC. Furthermore, three mediation models (individual, parallel multiple, and serial multiple) were utilized to investigate the mediating roles of -SPA1 and Kmax in the relationship between eye rubbing and KC.

**Results:**

After adjusting for confounding factors, the odds ratio and 95% confidence interval (CI) for the time of eye rubbing, -SPA1, and Kmax in relation to KC were 1.02 (1.01, 1.04), 1.16 (1.12, 1.19), and 3.86 (2.52, 5.92), respectively. The individual mediation model indicated that the indirect effects of -SPA1 and Kmax were 0.084 and 0.056, respectively. The parallel multiple mediation model showed a total indirect effect of 0.081 for -SPA1 and Kmax. Additionally, the serial multiple mediation model (time of eye rubbing → -SPA1 → Kmax → KC) indicated that following -SPA1, Kmax partially mediated the relationship between the time of eye rubbing and KC with a total indirect effect of 0.024 (95% CI: 0.016–0.042), accounting for 14.5% of the total effect (time of eye rubbing on KC), while no significant indirect effect was found for Kmax alone.

**Conclusions:**

The individual, parallel multiple, and serial multiple mediation analyses consistently demonstrated the mediating roles of -SPA1 and Kmax in linking the duration of eye rubbing to KC. Notably, the serial mediation pathway (time of eye rubbing → -SPA1 → Kmax → KC) exhibited a significant indirect effect. These findings confirm and complement the theoretical framework linking eye rubbing to KC, providing a reference for further exploration of the pathogenesis of KC.

## Introduction

Keratoconus (KC) is a progressive corneal ectatic disorder characterized by localized thinning, irregular astigmatism, and blurred vision that could ultimately lead to corneal blindness if left untreated (Ferrari and Rama, 2020; Mas Tur et al., 2017; Matthaei et al., 2017). Recent data suggest that the prevalence of KC in China exceeds 0.5% (Pan et al., 2014; Xu et al., 2012), surpassing the global average of 0.13% (Hashemi et al., 2020). This contributes substantially to the national burden of visual impairment, with approximately 12.5 million people suffering from poor vision in China (Rebenitsch et al., 2011). In addition to its social and economic impacts, KC impairs the quality of life of the patients because of progressive vision loss (Hashemi et al., 2018; Godefrooij et al., 2017), although timely interventions like corneal collagen crosslinking could ameliorate disease progression and improve the prognosis (Sandvik et al., 2015; Yang et al., 2024a). Given the significant consequences of this condition, elucidating the risk factors is of paramount importance.

The pathogenesis of KC is widely regarded as multifactorial, encompassing both genetic predisposition and environmental influences (Bykhovskaya and Rabinowitz, 2021), including ultraviolet light exposure (Kang et al., 2020), positive family history of KC (Cheng et al., 2022), and repeated mechanical trauma from habitual rubbing of the eyes (Hashemi et al., 2020; Debourdeau et al., 2022; Guo et al., 2023). Our previous research has demonstrated that the body mass index (BMI) and atopy not only have independent associations with KC but also exhibit possible interactive effects with eye rubbing (Yang et al., 2022; Ren et al., 2023a). Of these risk factors, eye rubbing has garnered particular interest as a modifiable behavior: if eye rubbing indeed exacerbates corneal deformation and disease progression, health education targeting this practice could serve as a non-invasive preventive measure (Guo et al., 2023; Henriquez et al., 2019). However, the manner in which eye rubbing translates into structural and biomechanical changes of the cornea remains insufficiently characterized.

Technological advancements now allow detailed *in vivo* evaluations of the corneal architecture. Corneal Visualisation Scheimpflug Technology (Corvis ST) (Ali et al., 2014; Gao et al., 2022) and Pentacam HR (De Bernardo et al., 2020; Gustafsson et al., 2023) can be used to quantify the biomechanical and topographic parameters associated with the severity of KC. In particular, the corneal stiffness parameter at the first applanation (SPA1) has emerged as a novel measure of corneal biomechanical stability, where a lower value indicates a softer and potentially more vulnerable cornea (Yang et al., 2020; Roberts et al., 2017; Xian et al., 2023). Our previous studies show that SPA1 not only aids in effective identification of KC but also decreases in value as the severity of the disease increases (Yang et al., 2020; Yang et al., 2019; Ren et al., 2023b). Similarly, the maximal corneal keratometry (Kmax; a robust topographic metric from Pentacam HR) is known to be elevated in advanced KC (Gustafsson et al., 2023; Ng et al., 2021; Achiron et al., 2022). Kmax is the most widely validated topographic marker for KC severity and is commonly regarded as an assessment metric for progression in meta-analyses (Ferdi et al., 2019). Although extant studies have advanced our understanding of the pathogenesis of KC, critical knowledge gaps persist. Mou et al. (2022) reported eye rubbing as a risk factor for KC based on studies in the east coast of China, but the underlying mechanism was not analyzed in depth. Mazharian et al. (2023) analyzed the association between eye rubbing intervention and KC progression by evaluating the impacts of eye rubbing and corneal topography parameters; however, this study did not involve an analysis of the impacts on corneal biomechanics. Previous studies have separately analyzed the relationships between eye rubbing and KC as well as the associations between corneal biomechanics and KC (Henriquez et al., 2019; Yang et al., 2020; Torres-Netto et al., 2022). However, a comprehensive evaluation of the potential causal relationships among eye rubbing, corneal biomechanical parameters, and topographic parameters in the development of KC is not available.

Mediation analysis refers to a causal inference framework that allows decomposition of the exposure–outcome relationships into direct and indirect effects transmitted through mediators (Carter et al., 2021; Liu et al., 2022). This method accounts for the interaction effects among exposures, mediators, and outcomes to enable robust identification of the mechanistic pathways in observational studies. Mediation models study the associations between exposures (X) and disease outcomes (Y) by introducing mediator variables (M). Mediation occurs when the effect of X on Y is transmitted through M. Statistically, the existence of a mediation effect can be tested using the value of the indirect path X→M→Y and its deviation from zero (Liu et al., 2022). Therefore, in the present case–control study nested within the Chinese keratoconus (CKC) cohort, we examined three factors: (1) association between eye rubbing and risk of KC; (2) how the corneal biomechanical (SPA1) and topographic (Kmax) parameters are related to KC; (3) mediating roles of SPA1 and Kmax in the pathway linking eye rubbing to KC. These findings are expected to clarify the mechanistic underpinnings of KC and inform usable behavioral or clinical strategies for disease prevention.

## Materials and methods

### Participants

The case–control analysis in the present study utilizes data from the CKC cohort study, which is a population-based longitudinal prospective cohort study, as detailed elsewhere (Yang et al., 2024b). Briefly, the CKC cohort study is an ongoing study focused on preventing KC progression and is a pioneering effort on understanding the effects of gene‒environment interactions on KC progression. The standards of diagnosis for KC used in present study were as follows (Yang et al., 2024b; Gomes et al., 2015): one or more positive signs upon slit-lamp examination (Vogt’s striae, Fleischer’s ring, Munson’s sign, or corneal scar), a Belin Ambrosio enhanced ectasia total deviation (BAD-D) index value ≥2.6, and an asymmetric bowtie pattern with or without skewed axes based on a corneal topography map. The inclusion criteria for the control group were as follows: the subjects were scheduled for refractive surgery with corneal astigmatism > -1.5 D, spherical equivalent > -8.0 D, corrected distance visual acuity in LogMAR ≤0.1, and normal corneal topography map. Based on the age (±3 years) and gender of the patients, comparable control individuals were matched to prevent bias in the present study. Finally, a total of 791 participants (395 KC patients and 396 control subjects) were recruited after excluding individuals who have had corneal surgery, trauma, history of contact lenses within 2 weeks, or missing data. The present study abides by the guidelines of the Declaration of Helsinki and was approved by the institutional review board of our institute (HNEECKY-2019 (5)). Written informed consent was obtained from each participant or their legal guardians/next of kin before participation in the study.

### Covariates

Information on the demographic characteristics and time of eye rubbing was collected through face-to-face interviews by trained personnel and in strict accordance with the standard operating manuals of the CKC cohort study (Yang et al., 2022; Yang et al., 2024b). The educational levels of the participants were divided into two groups as high school or above and middle school or below; the occupation types were classified into student and others; the duration of eye usage was recorded based on response to the question of how much time did you spend using paper products (books, newspapers, etc.) and electronic products (computer, cell phone, etc.) per day in the past week? The participant heights were obtained using a tape measure while leaning against a calibrated wall without wearing shoes. The individual weights were obtained using an Omron body fat body weight measurement device (V. BODY HBF-371, Omron, Japan). The time of eye rubbing for the subjects was assessed based on the following three questions (Ren et al., 2023a): How many days do you rub your eyes per week? How many times do you rub your eyes each day? How long do you rub your eyes each time? Then, the time of eye rubbing (s/d) was evaluated using the formula: time (s) × frequency (per day) × number of days (per week)/7.

### Parameters

The corneal topographic parameter Kmax that is widely used in the diagnosis and progression of KC was obtained using Pentacam HR (Oculus Optikgerate GmbH, Wetzlar, Germany). The corneal biomechanical parameter SPA1 was measured as force divided by displacement at the first applanation using Corvis ST (Oculus Optikgerate GmbH, Wetzlar, Germany). The spherical power, cylindrical power, and spherical equivalent value were also collected in the present study. To maintain consistency in the direction of association between the study parameters and KC, the inverse SPA1 (-SPA1) value was used in the current analysis.

### Statistical analysis

The normally distributed continuous variables were presented in terms of mean ± standard deviation (SD), while the non-normally distributed continuous variables were presented as median (Q1, Q3). The normally and abnormally distributed continuous variables between the KC and control groups were compared using Student’s t-test and Mann–Whitney test, respectively. The categorical variables were presented as numbers (percentage); the categorical variables between the KC and control groups were compared using the chi-squared or Fisher’s exact test.

The Pearson or Spearman correlation coefficient was used to calculate the correlations among the time of eye rubbing, -SPA1, Kmax, and KC depending on whether the data followed a normal distribution. The crude and adjusted models were developed to evaluate the associations among the time of eye rubbing, -SPA1, Kmax, and KC using generalized linear models. The odds ratio (OR) and 95% confidence interval (CI) were recorded by adjusting for age, gender, education level, occupation, history of eye disease, history of eye surgery, history of systemic diseases, family history of KC, duration of eye usage, and BMI.

In the present study, the influences of -SPA1 and Kmax on KC were evaluated through the direct and indirect effects using the individual, parallel multiple, and serial multiple mediation models. These three mediation models were structured using the “lavaan” R package (version 0.6–9) (Rosseel, 2012) to explore the mediating roles of -SPA1 and Kmax in eye rubbing and KC. The total effects, indirect effects, and mediated proportion of indirect effects for the three models were tested using 10,000 bootstrap samples to assess the statistical robustness. The 95% CI value was provided for the size of each effect, which did not include 0, indicating a significant moderating effect (Zhuang et al., 2023; Kim and Cha, 2022). The percentage of indirect effect out of the total effect represented the degree of indirect effect for the mediating factors. The subgroup analyses included the age subgroup (age ≤18 years vs. age >18 years), gender subgroup (male vs. female), and spherical equivalent group (spherical equivalent > −6.0 D vs. spherical equivalent ≤ −6.0 D) to explore the mediation effects of different corneal conditions on the results. All data were analyzed using R software version 4.4.0, and the statistical significance was set at two-tailed *p*-value <0.05.

## Results

### Study population characteristics

The case–control analysis included a total of 791 participants (KC cases: n = 395; controls: n = 396), whose baseline characteristics are detailed in Table 1. Compared to the controls, the KC cases demonstrated significantly higher values for the time of eye rubbing (median [Q1, Q3]: 6.00 [0.00, 30.00] vs. 0.00 [0.00, 0.00] min/d, *p* < 0.001), -SPA1 (mean ± SD: −60.66 ± 21.39 vs. −115.04 ± 16.51, *p* < 0.001), and Kmax (mean ± SD: 63.35 ± 11.57 D vs. 44.12 ± 1.66 D, *p* < 0.001). No significant between-group differences were observed for age, gender, education level, history of eye surgery, family history of KC, weight, and spherical power (all *p* > 0.05). Statistically significant differences (*p* < 0.05) were noted between groups for the other selected variables.

**TABLE 1:** Distributions of selected variables of the study participants.

Variables	Controls (n = 396)	KC (n = 395)	All (n = 791)	*p*-value
Age (years, mean ± SD)	20.99 ± 4.62	21.36 ± 5.49	21.18 ± 5.07	0.309^a^
Gender (n, %)				0.254^b^
Males	270 (68.18)	284 (71.90)	554 (70.04)	
Females	126 (31.82)	111 (28.10)	237 (29.96)	
Education level (n, %)				0.544^b^
Middle school or below	162 (40.91)	170 (43.04)	332 (41.97)	
High school or above	234 (59.09)	225 (56.96)	459 (58.03)	
Occupation (n, %)				<0.001^b^
Student	93 (23.48)	232 (58.73)	325 (41.09)	
Others	303 (76.52)	163 (41.27)	466 (58.91)	
Allergic history (n, %)				<0.001^b^
No	374 (94.44)	327 (82.78)	701 (88.62)	
Yes	22 (5.56)	68 (17.22)	90 (11.38)	
History of eye disease (n, %)				<0.001^b^
No	391 (98.74)	336 (85.06)	727 (91.91)	
Yes	5 (1.26)	59 (14.94)	64 (8.09)	
History of eye surgery (n, %)				0.242^b^
No	374 (94.44)	380 (96.20)	754 (95.32)	
Yes	22 (5.56)	15 (3.80)	37 (4.68)	
History of systemic diseases (n, %)				<0.001^b^
No	380 (95.96)	242 (61.27)	622 (78.63)	
Yes	16 (4.04)	153 (38.73)	169 (21.37)	
Family history of KC (n, %)				0.082^b^
No	396 (100)	392 (99.24)	788 (99.62)	
Yes	0 (0)	3 (0.76)	3 (0.38)	
Duration of eye usage (min, mean ± SD)	426.04 ± 277.08	548.37 ± 261.9	490.13 ± 275.91	<0.001^a^
Height (cm, mean ± SD)	172.85 ± 7.8	170.53 ± 8.62	171.74 ± 8.28	<0.001^a^
Weight (kg, mean ± SD)	63.56 ± 11.16	64.31 ± 12.63	63.92 ± 11.88	0.383^a^
BMI (kg/m^2^, mean ± SD)	21.19 ± 2.86	22.00 ± 3.45	21.58 ± 3.18	<0.001^a^
Spherical power (D, mean ± SD)	−4.58 ± 1.8	−4.88 ± 3.99	−4.73 ± 3.08	0.163^a^
Cylindrical power (D, mean ± SD)	−0.72 ± 0.68	−3.95 ± 2.5	−2.30 ± 2.43	<0.001^a^
Spherical equivalent (D, mean ± SD)	−5.27 ± 2.09	−8.58 ± 4.85	−6.88 ± 4.09	<0.001^a^
Time of eye rubbing (min/d, median (Q1, Q3))	0.00 (0.00, 0.00)	6.00 (0.00, 30.00)	0.00 (0.00, 10.00)	<0.001^c^
-SPA1	−115.04 ± 16.51	−60.66 ± 21.39	−91.84 ± 31.94	<0.001^a^
Kmax	44.12 ± 1.66	63.35 ± 11.57	52.21 ± 12.21	<0.001^a^

BMI, body mass index; KC, keratoconus; Kmax: maximal corneal keratometry; SD, standard deviation; -SPA1, inverse of the stiffness parameter at the first applanation.

^a^ Student’s t-test was used to compare the normally distributed continuous variables between KC and controls.

^b^ Chi-squared test or Fisher exact test was used to test the distributions of categorical variables between KC and controls.

^c^ Mann–Whitney test was used to compare non-normally distributed continuous variables between KC and controls.

### Exposure–outcome associations

**FIGURE 1:**
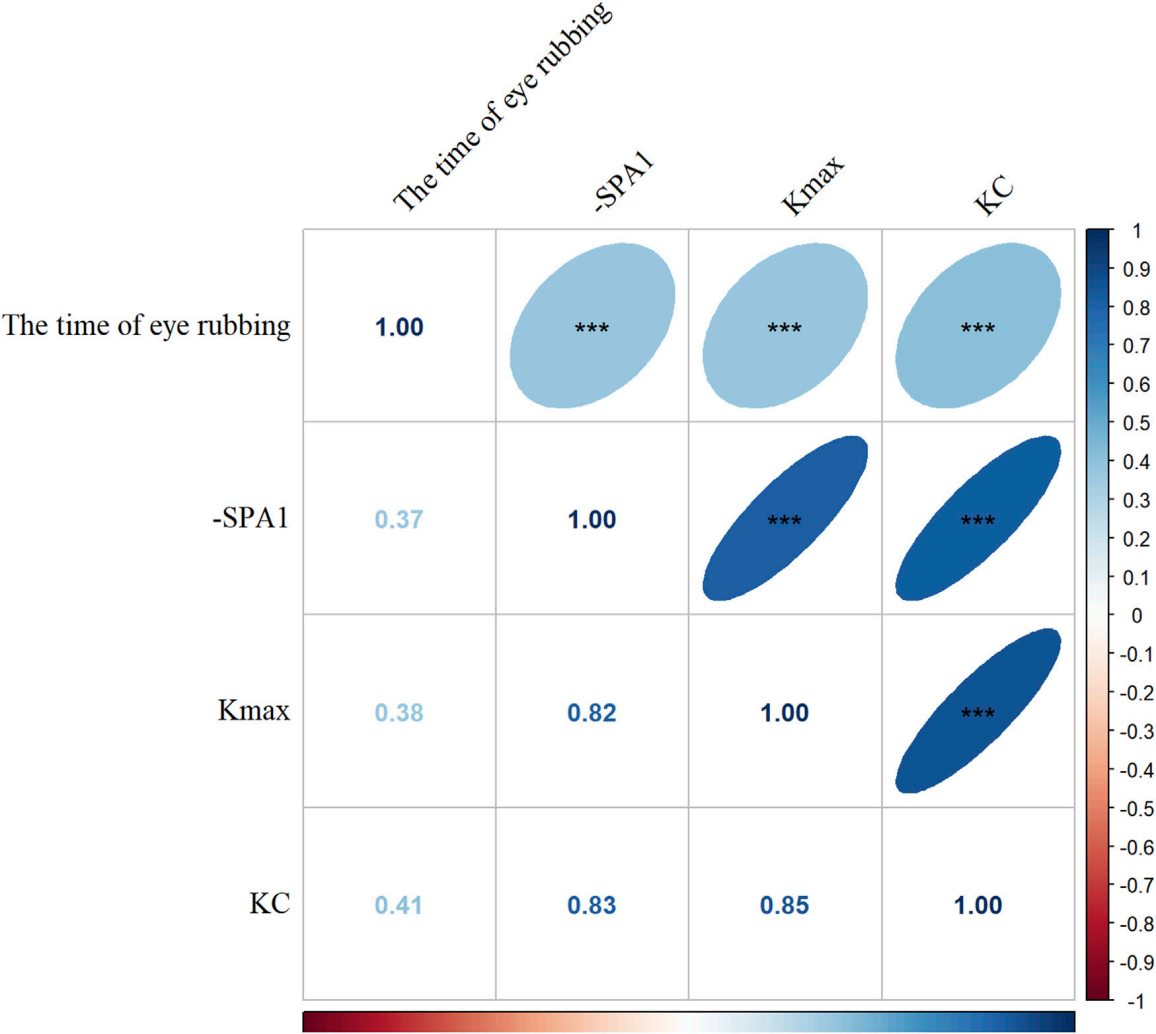
Correlation analysis among time of eye rubbing, inverse of the stiffness parameter at the first applanation (-SPA1), maximal corneal keratometry (Kmax), and keratoconus (KC). The Spearman correlation coefficients are shown (blue: positive correlation; red: negative correlation; darker color and flatter ellipse imply stronger correlation. ***p < 0.001).

**FIGURE 2:**
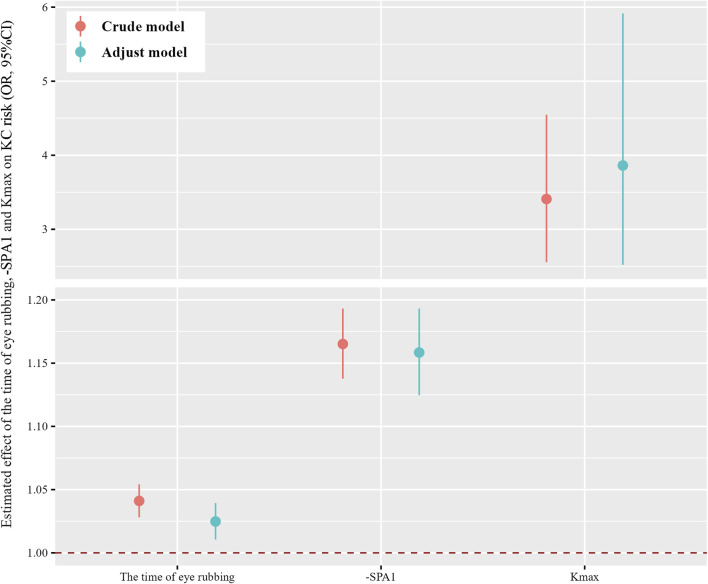
Estimated effects of the time of eye rubbing, -SPA1, and Kmax on risk of KC were analyzed using generalized linear models. The adjusted model considered age, gender, education level, occupation, history of allergy, history of eye disease, history of eye surgery, history of systemic diseases, family history of KC, duration of eye usage, and body mass index (BMI). The dots and lines show the odds ratios and corresponding 95% confidence intervals for the associations of the time of eye rubbing, -SPA1, and Kmax with KC.

Figure 1 demonstrates strong pairwise correlations among the time of eye rubbing, -SPA1, Kmax, and KC status (all *p* < 0.001). The generalized linear models (Figure 2) revealed dose-dependent relationships. For the crude model, the findings were as follows: time of eye rubbing (OR = 1.04 per min/d; 95% CI: 1.03–1.05); -SPA1 (OR = 1.17 per unit; 95% CI: 1.14–1.19); Kmax (OR = 3.41 per D; 95% CI: 2.56–4.55). For the model adjusted in terms of age, gender, education level, occupation, history of eye disease, history of eye surgery, history of systemic diseases, family history of KC, duration of eye usage, BMI, and spherical equivalent, the findings were as follows: time of eye rubbing (OR = 1.02 per min/d; 95% CI: 1.01–1.04); -SPA1 (OR = 1.16 per unit; 95% CI: 1.12–1.19); Kmax (OR = 3.86 per D; 95% CI: 2.52–5.92).

### Mediating effects of the biomechanical and topographic parameters

#### Individual mediation model

**FIGURE 3:**
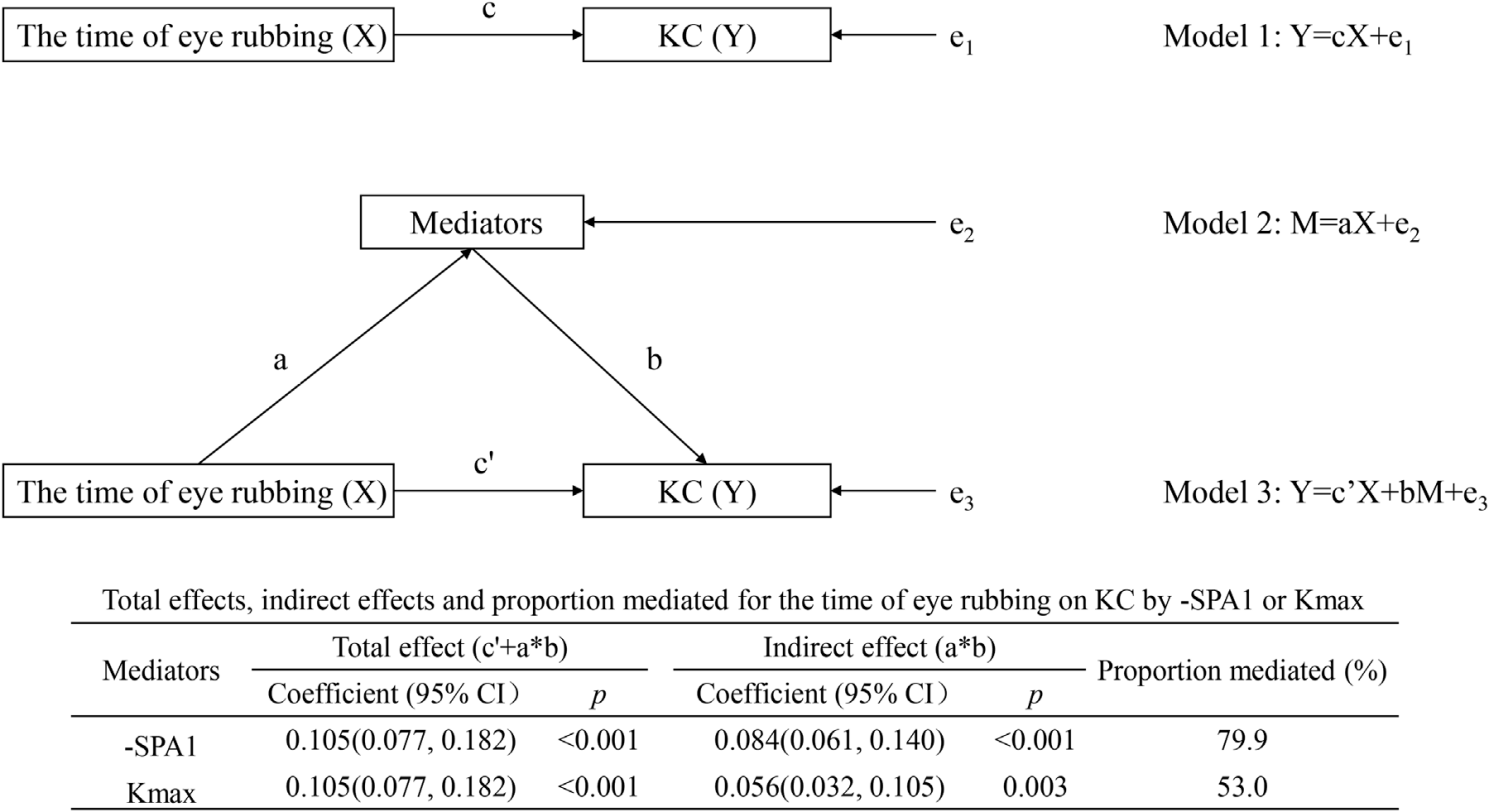
Individual mediation model showing the effects of time of eye rubbing on KC by -SPA1 or Kmax.

As shown in Figure 3, both -SPA1 and Kmax independently mediated the time of eye rubbing → -SPA1/Kmax → KC association. For the -SPA1 pathway, the indirect effect was 0.084 (95% CI: 0.061–0.140, *p* < 0.001), which accounted for 79.9% of the total effect; for the Kmax pathway, the indirect effect was 0.056 (95% CI: 0.032–0.105, *p* = 0.003), which explained 53.0% of the total effect.

#### Parallel multiple mediation model

**FIGURE 4:**
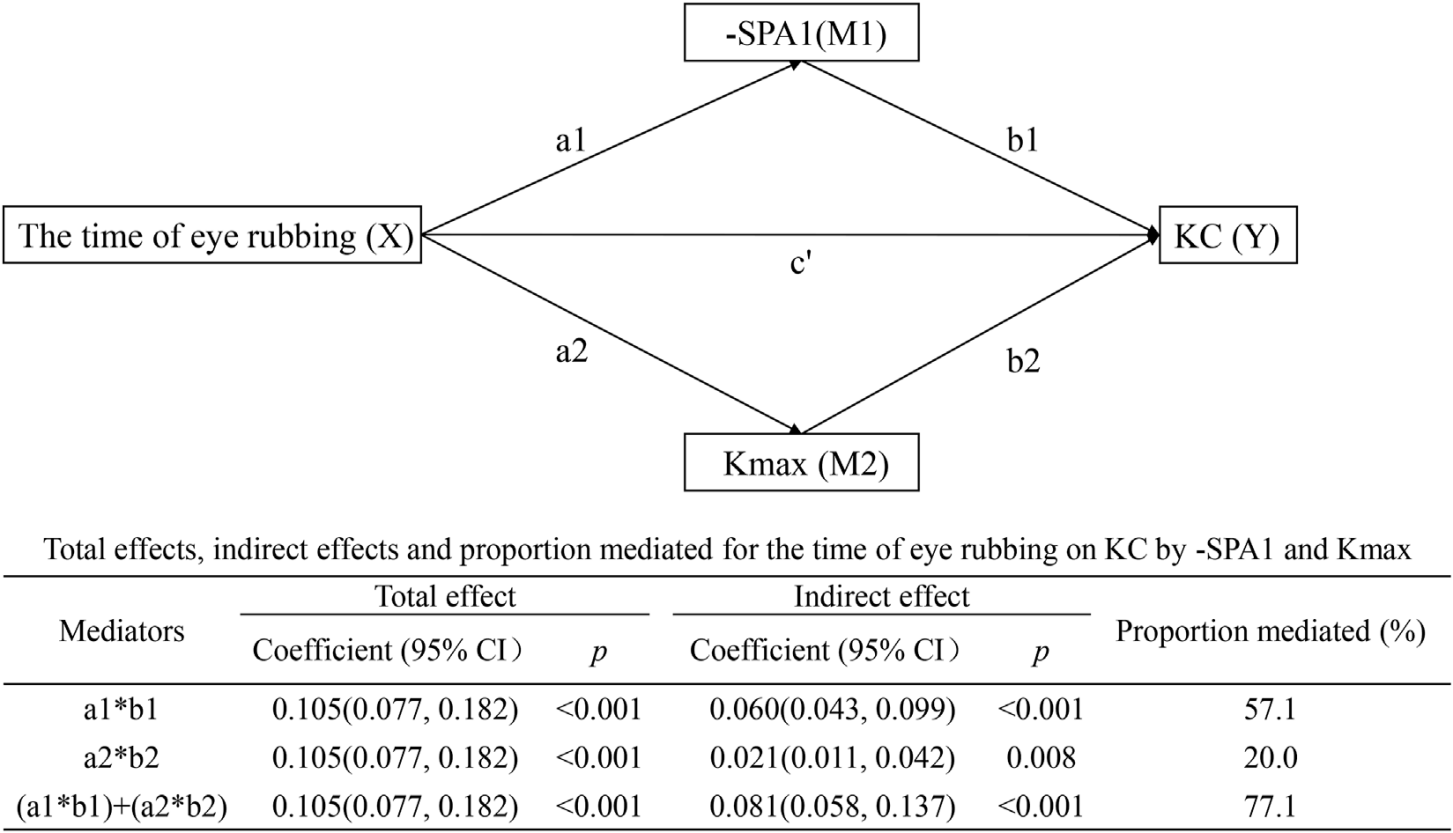
Parallel multiple mediation model showing the effects of time of eye rubbing on KC by -SPA1 and Kmax.

Figure 4 illustrates two concurrent pathways as follows: time of eye rubbing → -SPA1 → KC with effect = 0.060 (95% CI: 0.043–0.099, *p* < 0.001) and mediated proportion = 57.1%; time of eye rubbing → Kmax → KC with effect = 0.021 (95% CI: 0.011–0.042, *p* = 0.008) and mediated proportion = 20.0%. The combined indirect effect (0.081, 95% CI: 0.058–0.137, *p* < 0.001) accounted for 77.1% of the total association.

#### Serial multiple mediation model

**FIGURE 5:**
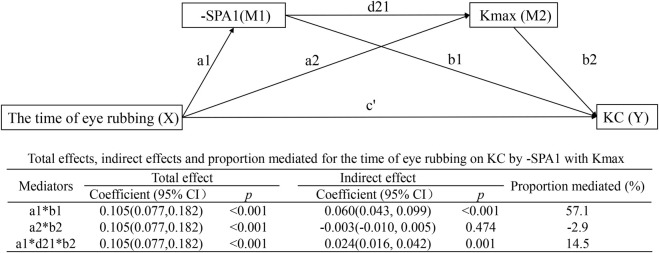
Serial multiple mediation model showing the effects of time of eye rubbing on KC by -SPA1 with Kmax.

Figure 5 demonstrates the interactions in the serial multiple mediation model as follows: time of eye rubbing → SPA1 → Kmax → KC with indirect effect = 0.024 (95% CI: 0.016–0.042, *p* = 0.001) and mediated proportion = 14.5%; time of eye rubbing → -SPA1 → KC with effect = 0.060 (95% CI: 0.043–0.099, *p* < 0.001) and mediated proportion = 57.1%. Notably, the direct path involving time of eye rubbing → Kmax → KC was non-significant (β = −0.003, 95% CI: −0.010–0.005, *p* = 0.474).

### Mediating effects in subgroup analyses

The indirect effect (time of eye rubbing → SPA1 → Kmax → KC) for the age >18 years subgroup was 0.019 (95% CI: 0.009–0.043, *p* = 0.036), while no significant effect was observed for the age ≤18 years subgroup (*p* = 0.072, Supplementary Figures S1–S6). Similarly, the indirect effect (time of eye rubbing → SPA1 → Kmax → KC) for the subgroup of male patients was 0.025 (95% CI: 0.014–0.063, *p* = 0.039), while no significant effect was observed for the subgroup of female patients (*p* = 0.054, Supplementary Figures S7–S12). In addition, the indirect effect (time of eye rubbing → SPA1 → Kmax → KC) for the spherical equivalent ≤ −6.0 D subgroup was 0.016 (95% CI: 0.009–0.034, *p* = 0.011), while no significant effect was noted for the spherical equivalent > −6.0 D subgroup (*p* = 0.501, Supplementary Figures S13–S18).

## Discussion

Technological advancements have enabled the identification of increasing numbers of patients with KC, highlighting the growing importance of exploring its etiology and pathogenesis (Zhang et al., 2024; Belin et al., 2022). The results reveal that the time of eye rubbing, -SPA1, and Kmax are all positively associated with KC. The three mediation models indicate that -SPA1 partially mediates the relationship between time of eye rubbing and KC. In the individual and parallel multiple mediation models, Kmax partially mediates the relationship between time of eye rubbing and KC, while no mediating effect is observed in the serial multiple mediation model. These findings suggest that increase in -SPA1 may lead to changes in Kmax, providing a reference for further exploration of the mechanisms of KC.

Currently, the etiology of KC still remains unclear (Ferrari and Rama, 2020; Mas Tur et al., 2017). Although genetic factors have been identified as contributors, environmental factors have also been found to play significant roles in the onset and development of KC (Hashemi et al., 2020). Furthermore, given the increasing numbers of cases with very asymmetrical KC and unilateral KC, experts have proposed that repeated mechanical trauma represented by eye rubbing could trigger the occurrence of KC (Henriquez et al., 2019; Saad et al., 2022). While occasional eye rubbing due to eye fatigue or waking is considered a benign activity, frequent or vigorous eye rubbing can have pathological consequences and cause damage to the cornea (Torres-Netto et al., 2022; McMonnies, 2008). In the present study, we observed a positive association between the time of eye rubbing and KC, which further supports the impact of eye rubbing on the development of KC. Although previous studies have shown that longer durations of eye rubbing increase the risk of KC (Mazharian et al., 2020; Dimacali et al., 2020; Shinzawa et al., 2019; Najmi et al., 2019), the specific effects of eye rubbing on KC remain to be fully understood. Emerging evidence shows that eye rubbing may induce corneal trauma (Rabinowitz et al., 2021), increase the corneal temperature (McMonnies, 2009), alter cone formation, and affect the corneal biomechanical stability (Torres-Netto et al., 2022; McMonnies et al., 2012; Gritz and McDonnell, 1988). These changes can traumatize the keratocytes and ultimately lead to inflammation, contributing to the pathogenesis of KC (Balasubramanian et al., 2013).

Corneal biomechanics refers to the ability of the cornea to undergo deformation in response to an external force and is a critical factor in determining the shape of the cornea (Kling and Hafezi, 2017). In the present case–control study, a positive relationship was found between -SPA1 and KC, which is consistent with the findings of previous studies (Flockerzi et al., 2022; Li et al., 2021; Xanthopoulou et al., 2023). Additionally, a recent study proved that the time of eye rubbing could alter SPA1 and make the cornea softer (Li et al., 2023). In an *ex vivo* model using enucleated porcine eyes, Torres-Netto et al. (2022) found that repetitive mechanical stresses could alter the corneal biomechanical properties and potentially trigger the progression of KC in predisposed corneas. Another study by Henriquez et al. (2019) reported a significant reduction in the intraocular pressure immediately after eye rubbing in eyes with KC. The present case–control study also shows that -SPA1 partially mediates the relationship between the time of eye rubbing and KC, which is consistent with the findings of previous studies that suggested that eye rubbing may cause KC by altering the biomechanical parameters (Henriquez et al., 2019; Torres-Netto et al., 2022; McMonnies et al., 2012). The mechanism by which eye rubbing alters the SPA1 level and induces KC may be explained as follows: first, eye rubbing could cause slippage of the corneal lamellae, leading to instantaneous reconstruction of the corneal collagen fibers and changes in the corneal biomechanical properties (Dawson et al., 2008); second, the corneal tissue has a certain degree of viscoelasticity, and eye rubbing may cause agitation as well as reduced viscosity (softening) of the cornea (McMonnies, 2009; Dawson et al., 2008); third, eye rubbing can increase the corneal temperature, which could reduce the bending resistance of the cornea (McMonnies, 2009); fourth, eye rubbing could cause cell flattening, chains of wing cells, cytoplasm leakage from the ruptured cells, displacement of the intercellular water from the rubbed area, and mucin formation (McMonnies, 2009). These changes could influence the mechanism by which eye rubbing alters SPA1 levels and contributes to the development of KC. Thus, the importance of education regarding eye rubbing and screening of corneal biomechanics needs to further emphasized in practical situations.

Corneal topographic parameters are widely used to detect the progression of KC (Mas Tur et al., 2017; Meyer et al., 2023). A study by Yousefi et al. (2020) proved that Kmax is one of the most effective topographic parameters for detecting KC. The current study also suggests that Kmax is positively related to KC, consistent with previous studies indicating that a higher Kmax value could be a risk factor for KC (Lenk et al., 2021; Kosekahya et al., 2018). In addition, our serial multiple mediation model suggests that Kmax plays a mediating role between eye rubbing and KC rather than being an independent factor. This mediation occurs through the pathway of eye rubbing → -SPA1 → Kmax → KC. Although more than half of the total effects of eye rubbing can be explained by -SPA1 and Kmax, our results are further supported by the causal serial mediation analysis, which shows significant path-specific effects involving -SPA1 and Kmax as causally ordered mediators. A previous study showed that abnormal biomechanical parameters can occur before topographic changes in KC patients (Dienes et al., 2014). Henriquez et al. (2019) also reported that KC eyes did not exhibit any statistical changes in the steeper or flattest anterior K after eye rubbing, which could be explained by the fact that KC eyes have weaker corneas. In addition, it has been reported that increased distending forces from eye rubbing may induce cone formation by curvature transfer of the fibrillar length from a diametrically opposite region of the cornea (McMonnies, 2009). This finding is consistent with the serial multiple mediation model and suggests that corneal biomechanics may alter the corneal topographic parameters in the development of KC, which provides a reference for exploring the pathogenesis of KC. In addition, the subgroup analyses provide critical insights into the consistency and variability of the mediating pathways linking eye rubbing to KC, particularly in relation to age, gender, and the spherical equivalent. These findings highlight both the robustness of the biomechanical mechanisms and potential effect modifications by the ocular structural factors. Future studies should therefore explore the potential interactions between eye rubbing and other risk factors in greater detail.

The present study primarily focuses on examining the relationships among eye rubbing, corneal biomechanics, corneal topographic parameters, and KC through three mediation models. However, some limitations should be noted. First, the information regarding eye rubbing was obtained through questionnaires in this study; although we followed standardized procedures and recorded the eye rubbing behaviors of patients via video, there may exist a recall bias that could affect the observed associations. It has been reported that the frequency and duration of eye rubbing tend to be underestimated when the behavior becomes a habit (Guo et al., 2023). McMonnies (2016) reported that the potential for underreporting rubbing activity may be greater in patients who have been advised by their practitioner or family members to not rub their eyes. Furthermore, people may not be fully aware of the extent of their rubbing behavior, which occurs unconsciously especially during sleep, and may accordingly underreport their rubbing activity (McMonnies, 2016). To enhance validity, future studies should implement objective measures such as wearable sensors to quantify eye-rubbing frequency to calibrate self-reported data against behavioral evidence and reduce recall bias. Second, all participants in this study were enrolled from a single tertiary care hospital, which could affect generalizability and be potentially confounding (e.g., atopic status and ocular surface disease). Although efforts were made to match the controls and patients based on age and gender, the extrapolation of the results could be affected and should be validated in a multicenter study. Third, the present study provides quantitative insights into the temporal dimension of eye rubbing (e.g., frequency and duration) but does not capture critical mechanical parameters such as rubbing intensity (force per unit area) or kinetic patterns (e.g., knuckle-mediated vs. fingertip-mediated rubbing); it is important to differentiate between these various types and techniques as it has been suggested that only repetitive and prolonged eye rubbing may alter the corneal biomechanics significantly to influence KC (Sahebjada et al., 2021). Hafezi et al. (2020) reported on the basis of high-precision balance that knuckle-type eye rubbing involves the application of significantly more force on the lids than the fingertip and fingernail types of rubbing; thus, future studies could focus on different aspects and integrate objective measures to deeply explore the effects on corneal biomechanics and KC. Lastly, although the present findings are based on a cross-sectional study and three mediation models to explore the roles of biomechanical and topographic parameters in eye rubbing and KC, it would be beneficial to include multicenter trials as well as prospective cohort studies with repeated corneal biomechanics measurements along with the inclusion of stronger objective measures of eye rubbing to ensure stable results.

## Conclusion

The results of this study indicate that the time of eye rubbing, -SPA1, and Kmax are positively associated with KC. The mediation models indicate that -SPA1 partially mediates the relationship between time of eye rubbing and KC and that the mediating effect of Kmax may be influenced by -SPA1. These findings confirm and complement the systemic theories of eye rubbing and KC, thereby providing a reference for exploring the mechanisms involved in KC.

## Data Availability

The original contributions presented in this study are included in the article/Supplementary Material, and any further inquiries may be directed to the corresponding authors.
