# Supplementary material for: Mediating roles of corneal biomechanical and topographic parameters in eye rubbing and keratoconus based on the Chinese keratoconus cohort study
Source: Front Bioeng Biotechnol. 2025 Jun 26;13:1595671. doi: 10.3389/fbioe.2025.1595671 (PMC12241165; doi:10.3389/fbioe.2025.1595671)
Supplement: Supplementary file 1 [file DataSheet1.pdf]

Supplementary Material

Mediation role of corneal biomechanical and topographic parameters in eye rubbing and keratoconus based on the Chinese keratoconus cohort study

Kaili Yang [1,2#], Runqi Tu [1#], Liyan Xu [1, 2], Yuwei Gu [1], Qi Fan [1], Shanshan Yin [1], Yi Yuan [3], Anqi Chang [4], Yifan Wang [3], Chenchen Yin [3], Yonghao Zang [5], Chenjiu Pang [1], Daniela Oehring [6], Yibin Hao [1]*, Shengwei Ren [1,2]*.

[1] Henan Provincial People’s Hospital, Henan Eye Hospital, People's Hospital of Zhengzhou University, Henan University People's Hospital, Zhengzhou, Henan, China.

[2] Eye Institute, Henan Academy of Innovations in Medical Science, Zhengzhou, Henan, China.

[3] Zhengzhou University People's Hospital, Henan Provincial People's Hospital, Henan Eye Hospital, Zhengzhou, Henan, China.

[4] Henan University People’s Hospital, Henan Provincial People’s Hospital, Henan Eye Hospital, Zhengzhou, Henan, China.

[5] Xinxiang Medical University, Henan Provincial People's Hospital, Henan Eye Hospital, Zhengzhou, Henan, China.

[6] School of Health and Human Sciences, University of Plymouth, Plymouth, UK

[#] Contributed equally to this work

***Correspondence:**

Shengwei Ren and Yibin Hao

E-mail: shengweiren1984@163.com and haoyibin0506@163.com

Supplementary Figures

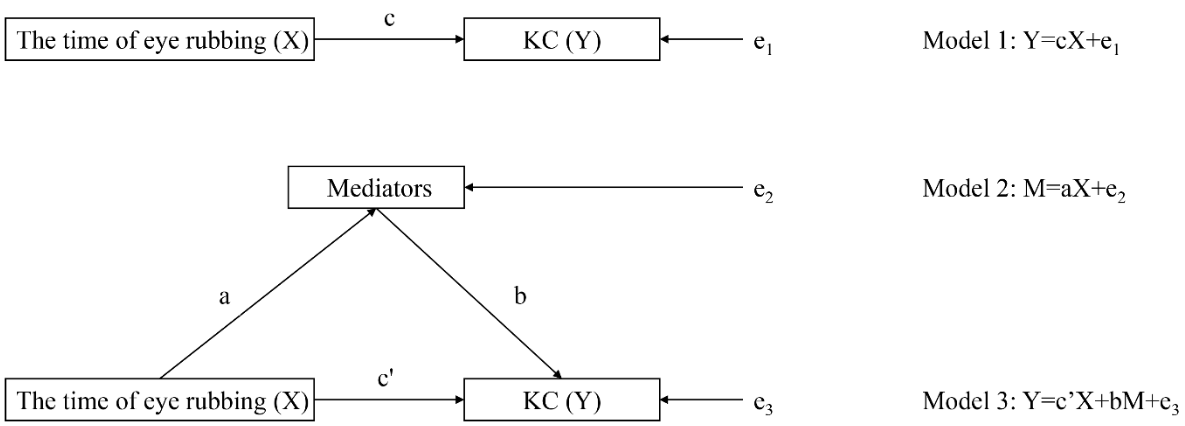

Total effects, indirect effects and proportion mediated for the time of eye rubbing on KC by -SPA1 or Kmax in age > 18 years old group

Mediators	Total effect (c'+a*b)		Indirect effect (a*b)		Proportion mediated (%)
	Coefficient (95% CI)	*p*	Coefficient (95% CI)	*p*	
-SPA1	0.094(0.063, 0.198)	0.007	0.068(0.034, 0.149)	0.024	72.3
Kmax	0.094(0.063, 0.198)	0.007	0.042(0.012, 0.129)	0.159	44.9

Supplemental Figure 1. The individual mediation model showing the time of eye rubbing on KC by -SPA1 or Kmax in age > 18 years old group.

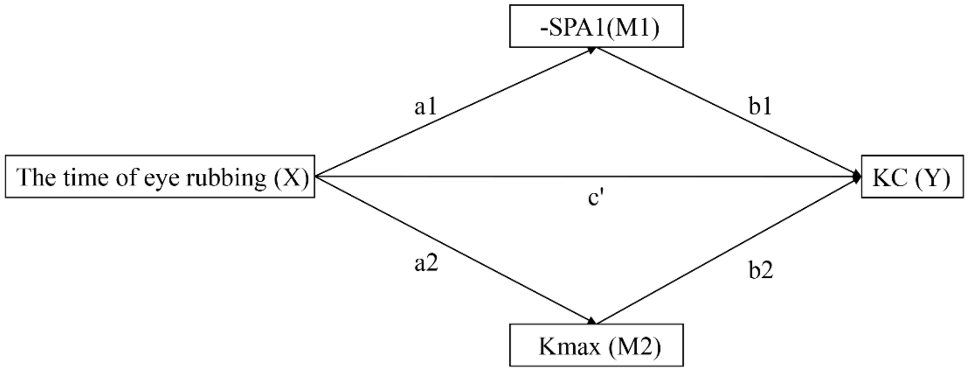

Total effects, indirect effects and proportion mediated for the time of eye rubbing on KC by -SPA1 and Kmax in age > 18 years old group

Mediators	Total effect		Indirect effect		Proportion mediated (%)
	Coefficient (95% CI)	*p*	Coefficient (95% CI)	*p*	
a1*b1	0.094(0.063, 0.198)	0.007	0.049(0.024, 0.110)	0.027	52.1
a2*b2	0.094(0.063, 0.198)	0.007	0.015(0.004, 0.048)	0.179	16.0
(a1*b1)+(a2*b2)	0.094(0.063, 0.198)	0.007	0.064(0.030, 0.154)	0.046	68.1

Supplemental Figure 2. The parallel multiple mediation model showing the time of eye rubbing on KC by -SPA1 and Kmax in age > 18 years old group.

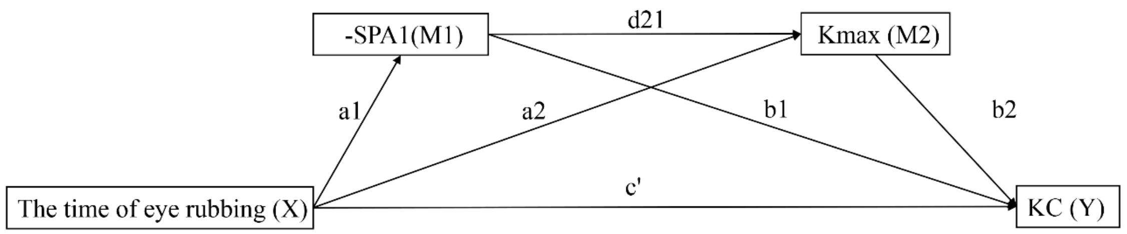

Total effects, indirect effects and proportion mediated for the time of eye rubbing on KC by -SPA1 with Kmax in age > 18 years old group

Mediators	Total effect		Indirect effect		Proportion mediated (%)
	Coefficient (95% CI)	*p*	Coefficient (95% CI)	*p*	
a1*b1	0.094(0.063,0.198)	0.007	0.049(0.024,0.110)	0.027	52.1
a2*b2	0.094(0.063,0.198)	0.007	-0.003(-0.010,0.007)	0.423	-3.2
a1*d21*b2	0.094(0.063,0.198)	0.007	0.019(0.009,0.043)	0.036	20.2

Supplemental Figure 3. The serial multiple mediation model showing the time of eye rubbing on KC by -SPA1 with Kmax in age > 18 years old group.

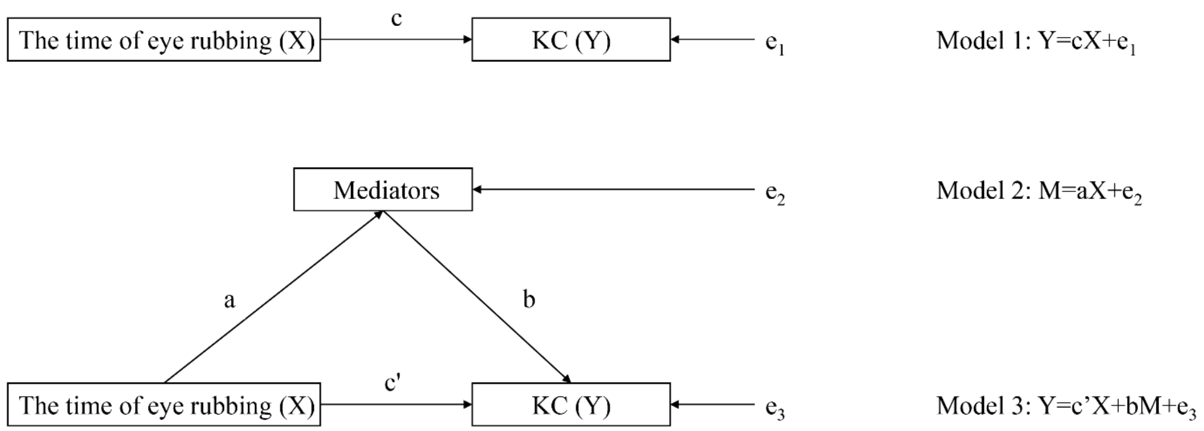

Total effects, indirect effects and proportion mediated for the time of eye rubbing on KC by -SPA1 or Kmax in age ≤ 18 years old group

Mediators	Total effect (c'+a*b)		Indirect effect (a*b)		Proportion mediated (%)
	Coefficient (95% CI)	*p*	Coefficient (95% CI)	*p*	
-SPA1	0.129(0.090,0.362)	0.084	0.106(0.082,0.250)	0.027	82.4
Kmax	0.129(0.090,0.362)	0.084	0.073(0.041,0.180)	0.086	56.6

Supplemental Figure 4. The individual mediation model showing the time of eye rubbing on KC by -SPA1 or Kmax in age ≤ 18 years old group.

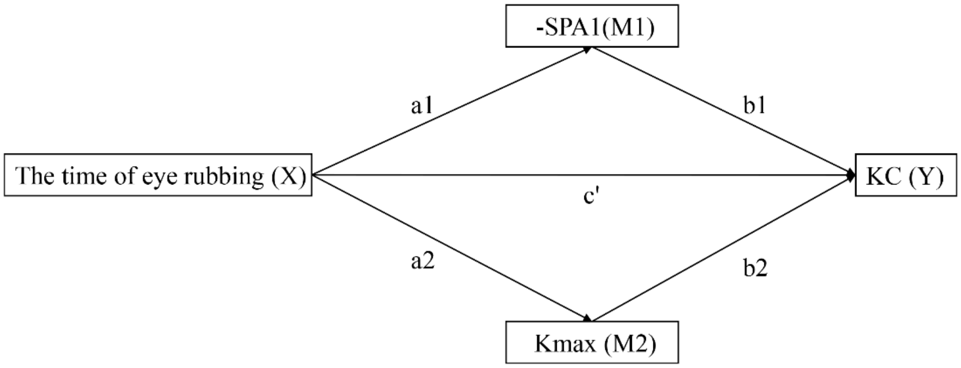

Total effects, indirect effects and proportion mediated for the time of eye rubbing on KC by -SPA1 and Kmax in age ≤ 18 years old group

Mediators	Total effect		Indirect effect		Proportion mediated (%)
	Coefficient (95% CI)	*p*	Coefficient (95% CI)	*p*	
a1*b1	0.129(0.090,0.362)	0.084	0.075(0.053,0.173)	0.027	58.1
a2*b2	0.129(0.090,0.362)	0.084	0.030(0.015,0.087)	0.146	23.3
(a1*b1)+(a2*b2)	0.129(0.090,0.362)	0.084	0.105(0.080,0.245)	0.036	81.4

Supplemental Figure 5. The parallel multiple mediation model showing the time of eye rubbing on KC by -SPA1 and Kmax in age ≤ 18 years old group.

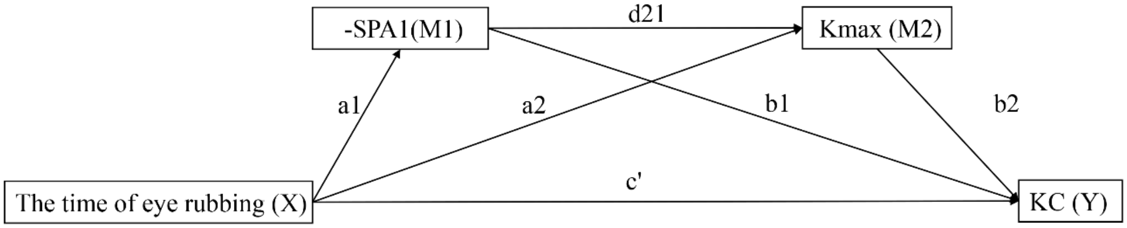

Total effects, indirect effects and proportion mediated for the time of eye rubbing on KC by -SPA1 with Kmax in age ≤ 18 years old group

Mediators	Total effect		Indirect effect		Proportion mediated (%)
	Coefficient (95% CI)	*p*	Coefficient (95% CI)	*p*	
a1*b1	0.129(0.090,0.362)	0.084	0.075(0.053,0.173)	0.027	58.1
a2*b2	0.129(0.090,0.362)	0.084	-0.002(-0.018,0.015)	0.860	-1.6
a1*d21*b2	0.129(0.090,0.362)	0.084	0.032(0.019,0.082)	0.072	24.8

Supplemental Figure 6. The serial multiple mediation model showing the time of eye rubbing on KC by -SPA1 with Kmax in age ≤ 18 years old group.

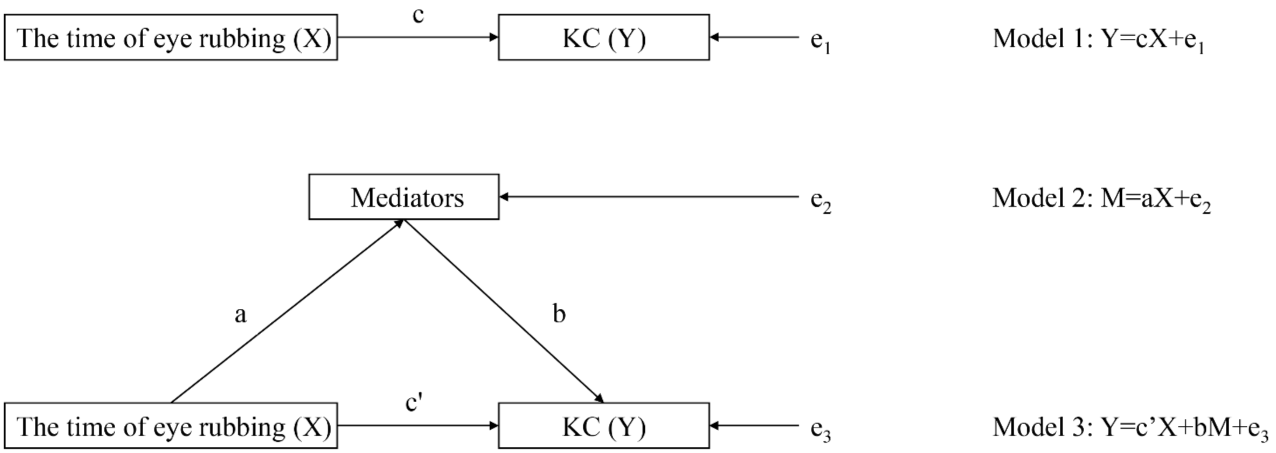

Total effects, indirect effects and proportion mediated for the time of eye rubbing on KC by -SPA1 or Kmax in males group

Mediators	Total effect (c'+a*b)		Indirect effect (a*b)		Proportion mediated (%)
	Coefficient (95% CI）	*p*	Coefficient (95% CI）	*p*	
-SPA1	0.105(0.071,0.282)	0.035	0.086(0.056,0.215)	0.021	81.1
Kmax	0.105(0.071,0.282)	0.035	0.064(0.032,0.177)	0.062	60.3

Supplemental Figure 7. The individual mediation model showing the time of eye rubbing on KC by -SPA1 or Kmax in males group.

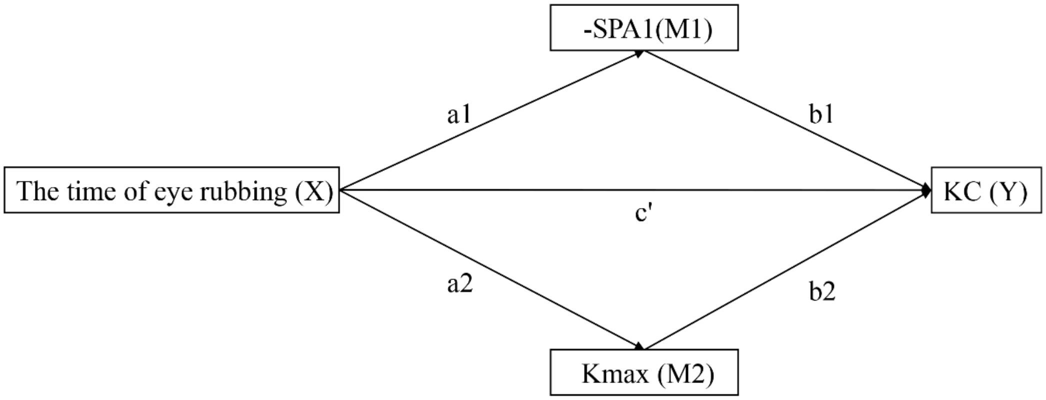

Total effects, indirect effects and proportion mediated for the time of eye rubbing on KC by -SPA1 and Kmax in males group

Mediators	Total effect		Indirect effect		Proportion mediated (%)
	Coefficient (95% CI）	*p*	Coefficient (95% CI）	*p*	
a1*b1	0.105(0.071,0.282)	0.035	0.061(0.039,0.151)	0.021	58.1
a2*b2	0.105(0.071,0.282)	0.035	0.025(0.012,0.071)	0.092	23.8
(a1*b1)+(a2*b2)	0.105(0.071,0.282)	0.035	0.086(0.054,0.223)	0.029	81.9

Supplemental Figure 8. The parallel multiple mediation model showing the time of eye rubbing on KC by -SPA1 and Kmax in males group.

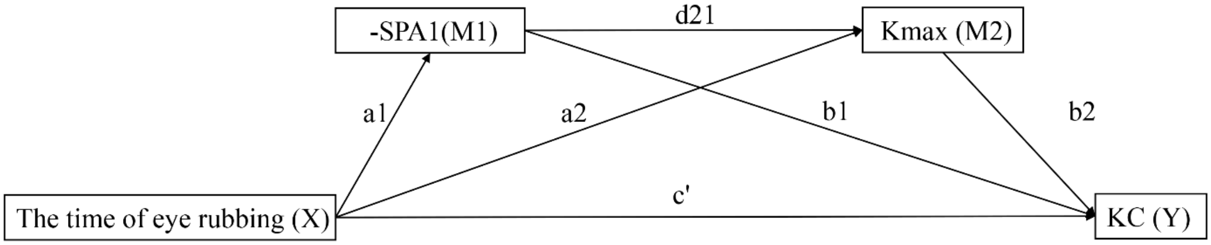

Total effects, indirect effects and proportion mediated for the time of eye rubbing on KC by -SPA1 with Kmax in males group

Mediators	Total effect		Indirect effect		Proportion mediated (%)
	Coefficient (95% CI）	*p*	Coefficient (95% CI）	*p*	
a1*b1	0.105(0.071,0.282)	0.035	0.061(0.039,0.151)	0.021	58.1
a2*b2	0.105(0.071,0.282)	0.035	0(-0.008,0.012)	0.942	0
a1*d21*b2	0.105(0.071,0.282)	0.035	0.025(0.014,0.063)	0.039	23.8

Supplemental Figure 9. The serial multiple mediation model showing the time of eye rubbing on KC by -SPA1 with Kmax in males group.

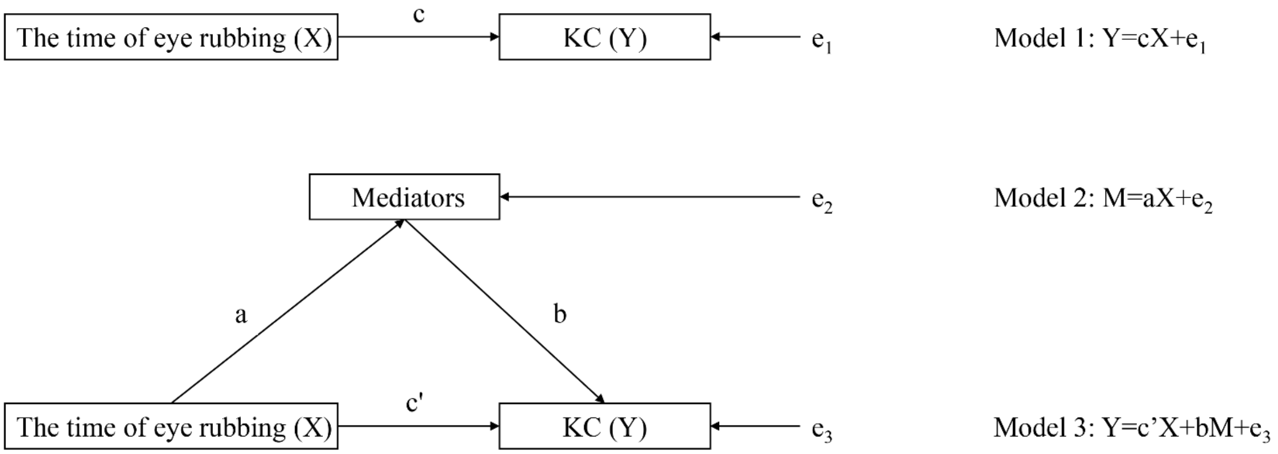

Total effects, indirect effects and proportion mediated for the time of eye rubbing on KC by -SPA1 or Kmax in females group

Mediators	Total effect (c'+a*b)		Indirect effect (a*b)		Proportion mediated (%)
	Coefficient (95% CI）	*p*	Coefficient (95% CI）	*p*	
-SPA1	0.105(0.072,0.262)	0.040	0.082(0.059,0.173)	0.009	77.6
Kmax	0.105(0.072,0.262)	0.040	0.039(0.013,0.129)	0.203	36.7

Supplemental Figure 10. The individual mediation model showing the time of eye rubbing on KC by -SPA1 or Kmax in females group.

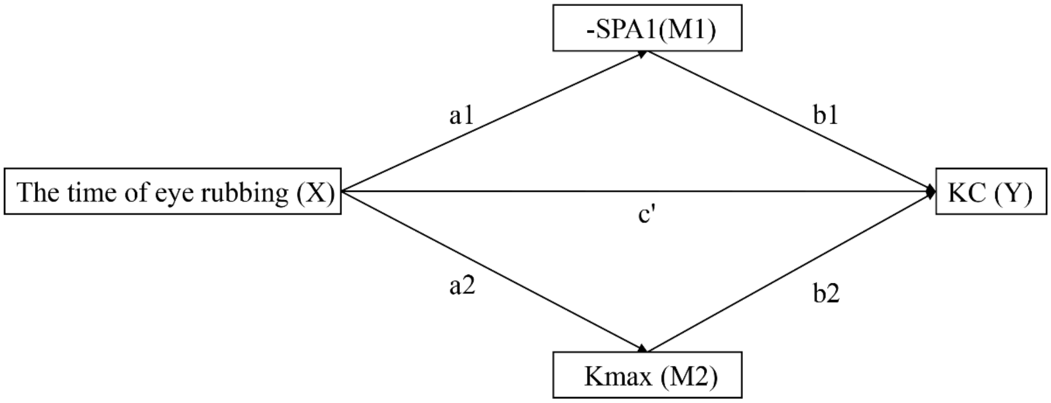

Total effects, indirect effects and proportion mediated for the time of eye rubbing on KC by -SPA1 and Kmax in females group

Mediators	Total effect		Indirect effect		Proportion mediated (%)
	Coefficient (95% CI）	*p*	Coefficient (95% CI）	*p*	
a1*b1	0.105(0.072,0.262)	0.040	0.059(0.040,0.127)	0.013	56.2
a2*b2	0.105(0.072,0.262)	0.040	0.014(0.003,0.049)	0.254	13.3
(a1*b1)+(a2*b2)	0.105(0.072,0.262)	0.040	0.073(0.053,0.170)	0.022	69.5

Supplemental Figure 11. The parallel multiple mediation model showing the time of eye rubbing on KC by -SPA1 and Kmax in females group.

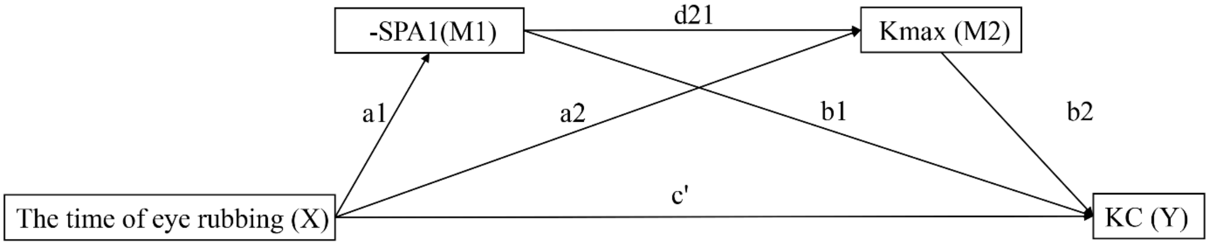

Total effects, indirect effects and proportion mediated for the time of eye rubbing on KC by -SPA1 with Kmax in females group

Mediators	Total effect		Indirect effect		Proportion mediated (%)
	Coefficient (95% CI）	*p*	Coefficient (95% CI）	*p*	
a1*b1	0.105(0.072,0.262)	0.040	0.059(0.040,0.127)	0.013	56.2
a2*b2	0.105(0.072,0.262)	0.040	-0.009(-0.024,0.004)	0.216	-8.6
a1*d21*b2	0.105(0.072,0.262)	0.040	0.023(0.010,0.055)	0.054	21.9

Supplemental Figure 12. The serial multiple mediation model showing the time of eye rubbing on KC by -SPA1 with Kmax in females group.

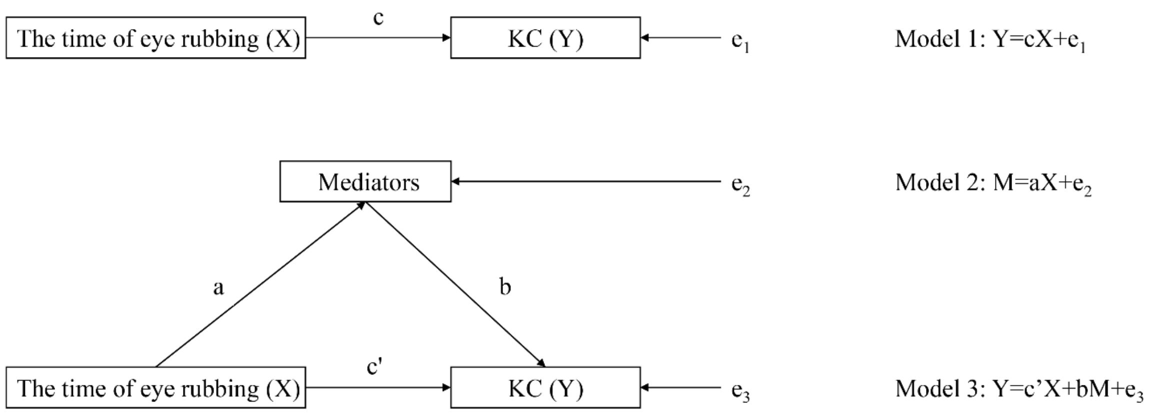

Total effects, indirect effects and proportion mediated for the time of eye rubbing on KC by -SPA1 or Kmax in spherical equivalent > -6 group

Mediators	Total effect (c'+a*b)		Indirect effect (a*b)		Proportion mediated (%)
	Coefficient (95% CI）	*p*	Coefficient (95% CI）	*p*	
-SPA1	0.102(0.073,0.602)	0.460	0.047(0.027,0.279)	0.483	45.5
Kmax	0.102(0.073,0.602)	0.460	0.039(0.015,0.305)	0.599	37.9

Supplemental Figure 13. The individual mediation model showing the time of eye rubbing on KC by -SPA1 or Kmax in spherical equivalent > -6.0 D group.

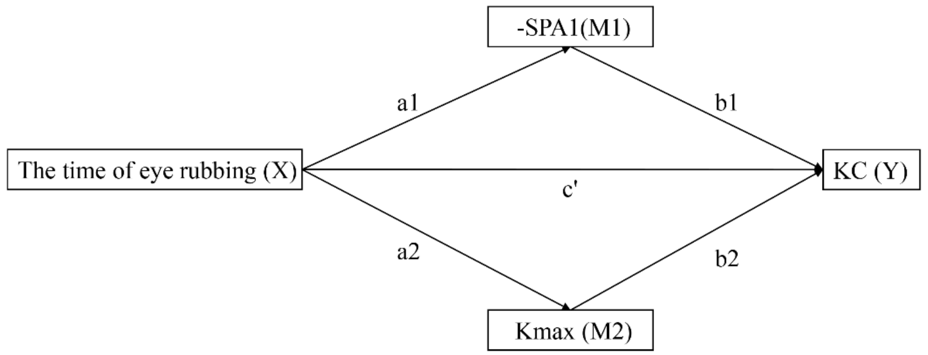

Total effects, indirect effects and proportion mediated for the time of eye rubbing on KC by -SPA1 and Kmax in spherical equivalent > -6 group

Mediators	Total effect		Indirect effect		Proportion mediated (%)
	Coefficient (95% CI）	*p*	Coefficient (95% CI）	*p*	
a1*b1	0.102(0.073,0.602)	0.460	0.027(0.014,0.168)	0.488	26.5
a2*b2	0.102(0.073,0.602)	0.460	0.021(0.008,0.166)	0.617	20.6
(a1*b1)+(a2*b2)	0.102(0.073,0.602)	0.460	0.048(0.024,0.328)	0.540	47.1

Supplemental Figure 14. The parallel multiple mediation model showing the time of eye rubbing on KC by -SPA1 and Kmax in spherical equivalent > -6.0 D group.

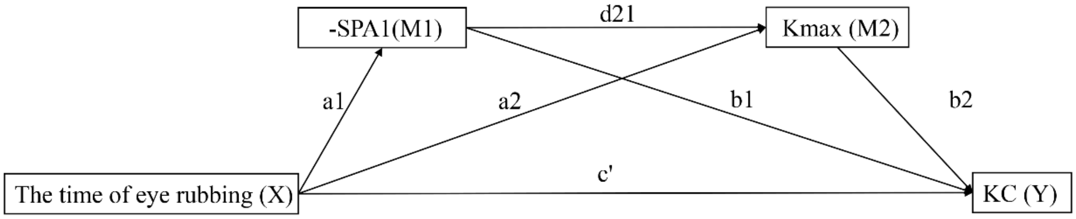

Total effects, indirect effects and proportion mediated for the time of eye rubbing on KC by -SPA1 with Kmax in spherical equivalent > -6 group

Mediators	Total effect		Indirect effect		Proportion mediated (%)
	Coefficient (95% CI）	*p*	Coefficient (95% CI）	*p*	
a1*b1	0.102(0.073,0.602)	0.460	0.027(0.014,0.168)	0.488	26.5
a2*b2	0.102(0.073,0.602)	0.460	0.002(-0.004,0.054)	0.914	2.0
a1*d21*b2	0.102(0.073,0.602)	0.460	0.019(0.010,0.117)	0.501	18.6

Supplemental Figure 15. The serial multiple mediation model showing the time of eye rubbing on KC by -SPA1 with Kmax in spherical equivalent > -6.0 D group.

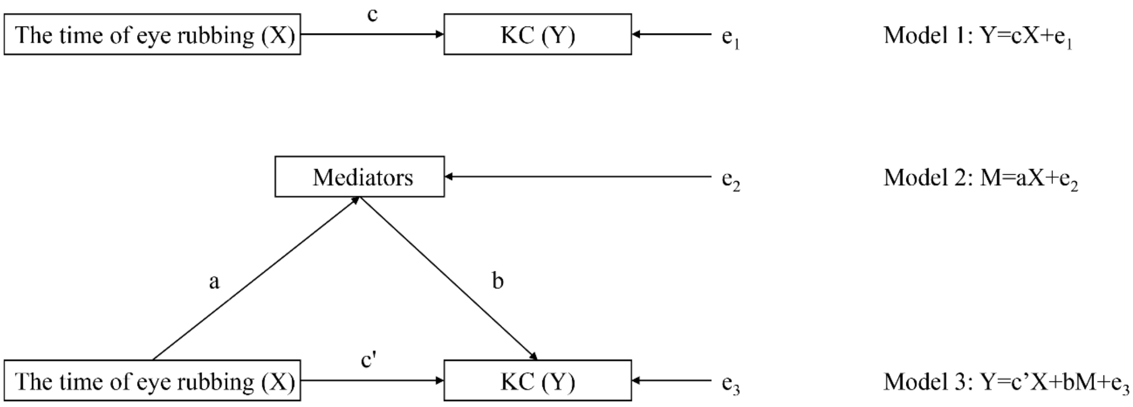

Total effects, indirect effects and proportion mediated for the time of eye rubbing on KC by -SPA1 or Kmax in spherical equivalent ≤ -6 group

Mediators	Total effect (c'+a*b)		Indirect effect (a*b)		Proportion mediated (%)
	Coefficient (95% CI）	*p*	Coefficient (95% CI）	*p*	
-SPA1	0.083(0.055,0.176)	0.007	0.088(0.066,0.162)	<0.001	105.5
Kmax	0.083(0.055,0.176)	0.007	0.050(0.021,0.111)	0.031	60.1

Supplemental Figure 16. The individual mediation model showing the time of eye rubbing on KC by -SPA1 or Kmax in spherical equivalent ≤ -6.0 D group.

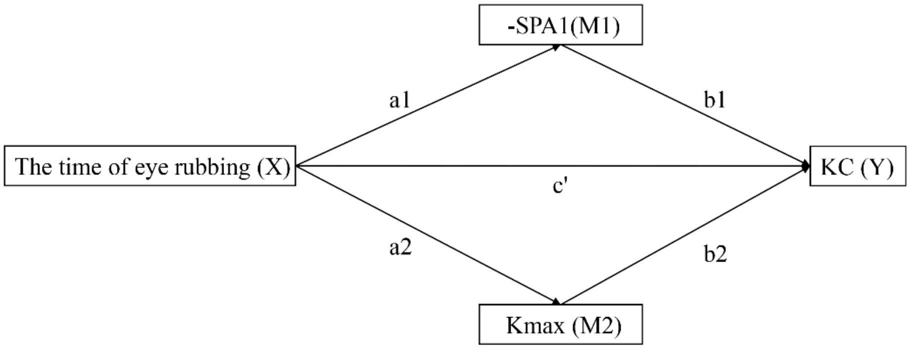

Total effects, indirect effects and proportion mediated for the time of eye rubbing on KC by -SPA1 and Kmax in spherical equivalent ≤ -6 group

Mediators	Total effect		Indirect effect		Proportion mediated (%)
	Coefficient (95% CI)	*p*	Coefficient (95% CI)	*p*	
a1*b1	0.083(0.055,0.175)	0.007	0.071(0.053,0.130)	<0.001	85.5
a2*b2	0.083(0.055,0.175)	0.007	0.013(0.005,0.033)	0.070	15.7
(a1*b1)+(a2*b2)	0.083(0.055,0.175)	0.007	0.085(0.062,0.159)	0.001	102.4

Supplemental Figure 17. The parallel multiple mediation model showing the time of eye rubbing on KC by -SPA1 and Kmax in spherical equivalent ≤ -6.0 D group.

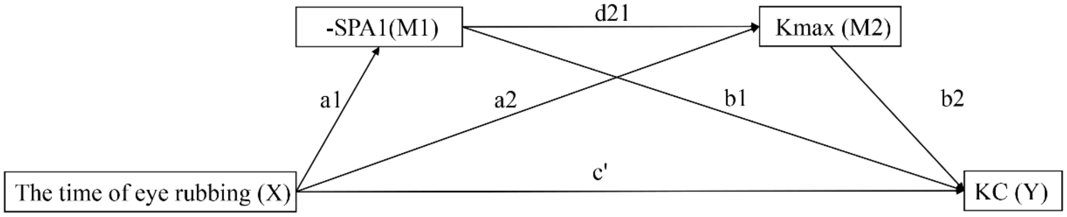

Total effects, indirect effects and proportion mediated for the time of eye rubbing on KC by -SPA1 with Kmax in spherical equivalent ≤ -6 group

Mediators	Total effect		Indirect effect		Proportion mediated (%)
	Coefficient (95% CI)	*p*	Coefficient (95% CI)	*p*	
a1*b1	0.083(0.055,0.175)	0.007	0.071(0.053,0.130)	<0.001	85.5
a2*b2	0.083(0.055,0.175)	0.007	-0.003(-0.012,0.005)	0.461	-3.6
a1*d21*b2	0.083(0.055,0.175)	0.007	0.016(0.009,0.034)	0.011	19.3

Supplemental Figure 18. The serial multiple mediation model showing the time of eye rubbing on KC by -SPA1 with Kmax in spherical equivalent ≤ -6.0 D group.
